# A survey of public attitudes towards third-party reproduction in Japan in 2014

**DOI:** 10.1371/journal.pone.0198499

**Published:** 2018-10-31

**Authors:** Naoko Yamamoto, Tetsuya Hirata, Gentaro Izumi, Akari Nakazawa, Shinya Fukuda, Kazuaki Neriishi, Tomoko Arakawa, Masashi Takamura, Miyuki Harada, Yasushi Hirota, Kaori Koga, Osamu Wada-Hiraike, Tomoyuki Fujii, Minoru Irahara, Yutaka Osuga

**Affiliations:** 1 Department of Obstetrics and Gynecology, Faculty of Medicine, University of Tokyo, Hongo, Bunkyo-ku, Tokyo, Japan; 2 Department of Obstetrics and Gynecology, Tokushima University Graduate School of Biomedical Sciences, Tokushima, Japan; Center For Human Reproduction, UNITED STATES

## Abstract

**Objective:**

The objective of this study was to examine public attitudes towards third-party reproduction and the disclosure of conception through third-party reproduction.

**Methods:**

We conducted the web-based survey for the public attitude towards third-party reproduction in February 2014. Twenty-five hundred people were recruited with equal segregation of age (20s, 30s, 40s, and 50s) and gender. We analyzed the association between gender, age, infertility, and ethical view using a questionnaire regarding donor sperm, donor oocyte, donor embryo, gestational surrogacy, and disclosure to offspring.

**Results:**

Of the respondents, 36.2% approved and 26.6% disapproved of gamete or embryo donation. The frequency of those who approved was lowest in females in the 50–59 year age group, and was significantly higher in males or females with infertility. Secondly, 40.9% approved and 21.8% disapproved of gestational surrogacy. The frequency of those who approved gestational surrogacy was higher in males or females with infertility. Thirdly, 46.3% of respondents agreed and 20.4% disagreed with “offspring have the right to know their origin”. Those who disagreed were primarily in the 50–59 year age group of both genders, and disagreement was significantly higher in the infertility group compared with non-infertility group.

**Conclusion:**

In this study, public attitudes were affected by gender, age, and experience of infertility. These study findings are important in understanding the attitude towards third-party reproduction and disclosure to the offspring. Respondents having indecisive attitudes were >30%, which might indicate an increased requirement for information and education to enhance the discussion on the ethical consensus on third-party reproduction in Japan.

## Introduction

The treatment cycle of assisted reproductive technology (ART) has dramatically increased; more than 1 million babies were born by ART between 2008 and 2010 [1]. In Japan, 424151 treatment cycles were carried out in 2015, and 51001 neonates (1 in 19.7 neonates born in Japan) were born [2]. Although ART is now widely accepted as clinically effective for treatment of many forms of subfertility, some people cannot conceive due to poor reproductive function, for example, uterine infertility and poor ovarian function. For those patients, ART using third party sperm, oocytes, or uterus is an option, known as third-party reproduction.

It is not technically difficult to perform third-party reproduction. Indeed, in the United States, there have been many cases of third-party reproduction, accounting for 16% of the total ART [3]. However, ethical concerns have been raised about these fertility treatments. The attitudes toward third-party reproduction are different in different countries, partly dependent on the legality of oocyte donation in a particular country. For example, in the European Union (EU), oocyte donation is permitted under certain conditions in France and Italy, but it is forbidden in Germany. In Japan, there is no legislation concerning ART, gamete or embryo donation, and gestational surrogacy, which have been controlled by the Japan Society of Obstetrics and Gynecology guidelines [4]. The academic society currently prohibits academic members from being involved in surrogate pregnancies, but sperm or egg donation is not prohibited and performed in limited numbers.

The ethical consequences of using donated gametes, embryos, and gestational surrogacy are a matter for debate among professionals and society in general. In Japan, there have been several surveys on general attitudes toward third-party reproduction in 1999, 2003, and 2007 [5–7]. Furthermore, the matter of disclosure of donor conception to donor offspring is a contentious issue [8]. Therefore, we sought to conduct a survey on public attitudes to third-party reproduction and disclosure of donor conception to offspring. In this study, the association between gender, age, infertility, and the ethical view were examined in a large representative sample thorough a web-based questionnaire regarding donor sperm, donor oocytes, donor embryos, gestational surrogacy, and disclosure of donor conception.

## Materials and methods

We conducted a web-based survey to assess public attitudes towards third-party reproduction. The sampling frame was developed by an internet research company and they sent out questionnaires through a website and collected the responses. Respondents were asked to read a summary page explaining the purpose and content of the questionnaire prior to starting the survey. In answering the questionnaire, reference materials (S1 Fig) were also included to deepen the understanding of respondents’ opinion on third-party reproduction. The questionnaire also included items regarding age, gender, marriage status, number of children, annual family income, and educational background (Supplemental Document 1). The web-based questionnaire was available online from 14^th^ February to 25^th^ February 2014. The program was such that only complete questionnaires could be submitted. Twenty-five hundred people were recruited with an equal segregation of age (20s, 30s, 40s, and 50s) and sex. We planned to collect 312 or 313 samples for each group of men and women in their 20s, 30s, 40s, and 50s. The upper limit of each sample collection was determined to be 375 samples. When the upper limit was reached, the collection of samples was closed and further samples were not obtained. For each group, 312 or 313 samples were randomly selected. Through this process, the internet research company extracted 2500 samples with equal segments of age and gender. Finally, we received the results of this extracted 2500 samples from the internet research company and subsequently used these for analysis. The study was conducted with the approval of the ethics committee of the University of Tokyo Hospital.

### Statistical analysis

The statistical analyses were performed using JMP pro version 13 (SAS Institute Inc, Cary, NC, USA). The categorical data were analyzed using chi-square test and Fisher’s exact test, and were presented as numbers and percentages. A P value < 0.05 was considered statistically significant.

## Results

### Characteristics of respondents

We sent out the questionnaire to 8900 people and 2605 responded. The response rate was 29.3%. We randomly extracted 2500 samples with equal segments of age and gender. The study thus consisted of a total of 2500 respondents with an equal number across each age group (20s, 30s, 40s, and 50s). Answers from equal numbers of men and women in each age group were obtained. Of the respondents, 44.3% were unmarried and 55.7% were married. Survey respondents had a wide range of family income levels. The characteristics of the respondents are shown in Table 1 and S1 Table.

**Table 1:** Demographic data of respondents (n = 2500).

	Total	Male	Female
Age (years)			
20–29	624 (25.0%)	312 (25.0%)	312 (25.0%)
30–39	626 (25.0%)	313 (25.0%)	313 (25.0%)
40–49	626 (25.0%)	313 (25.0%)	313 (25.0%)
50–59	624 (25.0%)	312 (25.0%)	312 (25.0%)
Marital status			
Unmarried	1108 (44.3%)	649 (51.9%)	459(36.7%)
Married	1392 (55.7%)	601(48.1%)	791(63.3%)
Number of children			
0	1343 (53.7%)	740 (59.2%)	603 (48.2%)
1	407 (16.3%)	170 (13.6%)	237 (19.0%)
2	561 (22.4%)	249 (19.9%)	312 (25.0%)
3	164 (6.6%)	79 (6.3%)	85 (6.8%)
4	21 (0.8%)	11 (0.9%)	10 (0.8%)
more than 4	4 (0.2%)	1 (0.1%)	3 (0.2%)
Annual family income (yen/year)			
less than 1,000,000	145 (5.8%)	81(6.5%)	64 (5.1%)
1,000,000–3,000,000	412 (16.5%)	185 (14.8%)	227 (18.2%)
3,000,000–5,000,000	686 (27.4%)	345 (27.6%)	341 (27.3%)
5,000,000–7,000,000	571 (22.8%)	276 (22.1%)	295 (23.6%)
7,000,000–10,000,000	417 (16.7%)	223 (17.8%)	194 (15.5%)
10,000,000–15,000,000	200 (8.0%)	109 (8.7%)	91 (7.3%)
15,000,000-	69 (2.8%)	31 (2.5%)	38 (3.0%)
Education			
Junior high school	68 (2.7%)	36 (2.9%)	32 (2.6%)
High school	645 (25.8%)	308 (24.6%)	337 (27.0%)
Technical college	367 (14.7%)	177 (14.2%)	190 (15.2%)
Junior college	275 (11.0%)	16 (1.3%)	259 (20.7%)
University or graduate school	1144 (45.8%)	713 (57.0%)	431 (34.5%)
Others	1 (0.0%)	0 (0.0%)	1 (0.1%)
Experience of infertility			
Yes	358 (14.3%)	122 (9.8%)	236 (18.9%)
None	2142 (85.7%)	1128 (90.2%)	1014 (81.1%)
Experience of infertility treatment			
None	2259 (90.4%)	1166 (93.3%)	1093 (87.4%)
timed intercourse	206 (8.2%)	67 (5.4%)	139 (11.1%)
IUI (intrauterine insemination)	77 (3.1%)	24 (1.9%)	53 (4.2%)
IVF-ET (In vitro fertilization—embryo transfer)	44 (1.8%)	17 (1.4%)	27 (2.2%)
ICSI (intracytoplasmic sperm injection)	31 (1.2%)	13 (1.0%)	18 (1.4%)

### Social acceptance

Regarding social and ethical acceptance of gamete or embryo donation, the rate was 36.2% for “should be approved”, 26.6% for “should *not* be approved”, and 37.3% for “indecisive” (Table 2). The frequency of respondents who answered “should be approved” in males and females in the 20–29, 30–39, and 40–49 age groups was significantly higher than females in the 50–59 year age group. The frequency of respondents who answered “should *not* be approved” in males and females in the 50–59 year age group was significantly higher than in the other age groups. As shown in Table 3, the frequency of a “positive response” was higher in males or females with infertility than those without infertility, and indecisive responses were also less frequent in this group. There were no effects of marital status, number of children, or annual family income.

**Table 2:** Comparison of public attitude towards gamete or embryo donation and gestational surrogacy divided by age and gender.

				gamete donation, embryo donation			gestational surrogacy		
sex	age	group	N	should be approved	should *not* be approved	indecisive	should be approved	should *not* be approved	indecisive
		total	2500	36.2%	26.6%	37.3%	40.9%	21.8%	37.3%
male	20–29	A	312	38.1% ^H^	20.8%	41.0% ^B,D^	41.3%	17.6%	41.0% ^B,D^
	30–39	B	313	39.9% ^H^	27.2%	32.9%	41.9%	25.2% ^A^	32.9%
	40–49	C	313	34.2% ^H^	26.8%	39.0% ^D^	40.9%	20.1%	39.0% ^D^
	50–59	D	312	33.7%	35.6% ^A,B,C,E,F,G^	30.8%	40.4%	28.8% ^A,C,F,G^	30.8%
female	20–29	E	312	38.1% ^H^	23.1%	38.8%^D^	39.4%	21.8%	38.8%^D^
	30–39	F	313	42.5% ^C,D,H^	21.4%	36.1%	45.4%^H^	18.5%	36.1%
	40–49	G	313	36.1%^H^	23.3%	40.6%	40.6%	18.8%	40.6%
	50–59	H	312	26.6%	34.3% ^A,C,E,F,G^	39.1%^D^	37.2%	23.7%	39.1%^D^

^A,B,C,D,E,F,G,H^ denotes significantly high compared to group A, B, C, D, E, F, G, or H respectively (P<0.05).

**Table 3:** Comparison of public attitudes towards gamete or embryo donation and gestational surrogacy divided by fertility.

				gametes donation, embryo donation			gestational surrogacy		
sex	infertility	group	N	should be approved	should *not* be approved	indecisive	should be approved	should *not* be approved	indecisive
		total	2500	36.2%	26.6%	37.3%	40.9%	21.8%	37.3%
male	+	A	122	45.1% ^B,D^	32.8%	22.1%	50.8% ^B,D^	27.0%	22.1%
	-	B	1128	35.5%	27.0%	37.4% ^A,C^	40.1%	22.5%	37.4% ^A,C^
female	+	C	236	44.9% ^B,D^	27.1%	28.0%	51.7% ^B,D^	20.3%	28.0%
	-	D	1014	33.7%	25.1%	41.1% ^A,C^	38.1%	20.8%	41.1% ^A,C^

^A,B,C,D^ denotes significantly high compared to group A, B, C, or D respectively (P<0.05).

With respect to surrogacy, 40.9% of respondents agreed that it “should be approved”, 21.8% responded with “should *not* be approved”, and 37.3% were “indecisive” (Table 2). Overall, around 40% approved gestational surrogacy in any age or gender group, while the highest disapproval rate was in males in the 50–59 year age group. As shown in Table 3, the frequency of those who approved gestational surrogacy was higher in males or females with infertility than in those without infertility, which was similar to the attitudes towards gamete or embryo donation.

Next, we asked the question “who is eligible to be a recipient of gametes or embryos?” In response, couples, fact-married couples, single women, single men, or homosexual couples were regarded as acceptable recipients by 88.6%, 33.6%, 13.4%, 10.0%, and 25.8%, respectively (Table 4). The frequency of those who answered that it was acceptable for a homosexual couple was higher in females in the 20–29, 30–39, and 40–49 age groups than in males and females in the 50–59 year age group. There were no effects of marital status, number of children, annual family income, or infertility.

**Table 4:** Who is eligible to be a gamete or embryo recipient?

sex	age	group	N	married couple	fact-married couple, cohabiting couple	single woman	single man	homosexual couple	others	indecisive
total			904	88.6%	33.6%	13.4%	10.0%	25.8%	1.0%	7.5%
male	20–29	A	119	83.2%	31.9%	14.3%	13.4%	22.7%	0.0%	10.1%
	30–39	B	125	87.2%	31.2%	11.2%	8.8%	14.4%	1.6%	9.6%
	40–49	C	107	85.0%	29.0%	11.2%	9.3%	17.8%	1.9%	12.1%
	50–59	D	105	92.4%	41.9%	10.5%	9.5%	18.1%	0.0%	4.8%
female	20–29	E	119	88.2%	28.6%	13.4%	5.0%	38.7% ^A,B,C,D,H^	0.8%	5.9%
	30–39	F	133	90.2%	31.6%	18.0%	12.8%	36.8% ^A,B,C,D,H^	0.8%	6.0%
	40–49	G	113	94.7%	42.5%	16.8%	13.3%	34.5% ^B,C,D,H^	1.8%	4.4%
	50–59	H	83	88.0%	33.7%	9.6%	6.0%	19.3%	1.2%	7.2%

^A,B,C,D,E,F,G,H^ denotes significantly high compared to group A, B, C, D, E, F, G, or H respectively (P<0.05).

The most frequent reasons why respondents thought that gamete or embryo donation was socially acceptable were: "because there is a possibility that a person who cannot conceive due to disease can have a child" (79.6%), and "because people who are unable to conceive because of aging will be able to be pregnant"(43.9%) (S2 Table). In addition, the most frequent reasons why respondents thought that gamete or embryo donation was *not* socially acceptable were: “because there is no genetic link with parents” (46.8%), and “parent-child relationship will become unnatural” (39.9%) (S3 Table). As for surrogacy, the reasons why respondents thought that gestational surrogacy was socially acceptable were: “because there is a possibility that a person who cannot conceive due to illness can have a child” (76.7%), and “because there is a possibility that a woman whose uterus was removed due to disease or accident can have a child” (66.6%) (S4 Table). Furthermore, the reasons why respondents thought that gestational surrogacy was *not* socially acceptable were “parent-child relationship will become unnatural” (33.9%), and “pregnancy should be natural” (32.8%) (S5 Table).

### Personal opinion

To obtain a personal opinion, we asked whether respondents would choose to receive each type of third-party reproduction, assuming that they could not conceive in all other ways. For third-party reproduction overall, 1–3% would use it across all age groups, with no significant difference observed between each group. The frequency of those who would want to receive donor sperm, egg, embryo, or gestational surrogacy if their spouse wished to was significantly higher in males than in females (P < 0.01). Those who did not want to use third party reproduction was significantly lower in males than in females (P<0.01). The rate of those who would want to use donor sperm if their spouse wished to was 33.3%, 30.7%, 23.6%, and 22.4% in males in the 20–29, 30–39, 40–49, and 50–59 year age groups, respectively; and 26.6%, 21.4%, 15.3%, and 11.9% in females in the 20–29, 30–39, 40–49, and 50–59 year age groups, respectively, showing a decreasing tendency with age. The lowest frequency of those who would want to use third party reproduction if their spouse wished to was in females in the 50–59 year age group. On the other hand, the rate of those who did not want to use donor sperm was 64.4%, 66.8%, 75.4%, and 76.3% in males in the 20–29, 30–39, 40–49, and 50–59 year age groups, respectively, and 72.1%, 77.0%, 82.4%, and 86.5% in females in the 20–29, 30–39, 40–49, and 50–59 year age groups, respectively, showing an increasing tendency with age. These trends were common in the donor egg, donor embryo, and gestational surrogacy categories, as shown in Table 5. On the other hand, there was no significant difference in attitude towards gamete or embryo donation and surrogate pregnancy between the respondents with or without infertility of either gender (Table 6).

**Table 5:** Attitudes towards each type of third-party reproduction assuming the respondents cannot conceive in other ways.

				donor sperm			donor egg			donor embryo			gestational surrogacy		
sex	age	group	N	want to use	want to use, if my spouse wishes	not want to use, even if my spouse wishes	want to use	want to use, if my spouse wishes	not want to use, even if my spouse wishes	want to use	want to use, if my spouse wishes	not want to use, even if my spouse wishes	want to use	want to use, if my spouse wishes	not want to use, even if my spouse wishes
		Total	2500	1.7%	23.2%	75.1%	1.6%	25.2%	73.2%	1.6%	21.4%	76.9%	2.6%	26.5%	70.9%
male	20–29	A	312	2.2%	33.3% ^C,D,F,G,H^	64.4%	1.3%	38.1% ^D,E,F,G,H^	60.6%	1.9%	35.3% ^C,D,E,F,G,H^	62.8%	2.6%	41.7% ^C,D,E,F,G,H^	55.8%
	30–39	B	313	2.6%	30.7% ^D,F,G,H^	66.8%	2.2%	36.1% ^D,E,F,G,H^	61.7%	2.6%	33.5% ^C,D,E,F,G,H^	63.9%	3.2%	37.4% ^D,E,F,G,H^	59.4%
	40–49	C	313	1.0%	23.6% ^G,H^	75.4% ^A,B^	1.6%	31.0% ^E,F,G,H^	67.4%	1.0%	25.9% ^F,G,H^	73.2% ^A,B^	1.9%	31% ^F,G,H^	67.1% ^A^
	50–59	D	312	1.3%	22.4% ^G,H^	76.3% ^A,B^	1.6%	26.8% ^G,H^	71.2% ^A,B^	1.6%	23.4% ^F,G,H^	75.0% ^A,B^	1.6%	26.6% ^G,H^	71.8% ^A,B^
female	20–29	E	312	1.3%	26.6% ^G,H^	72.1% ^A^	1.6%	21.8% ^H^	76.6% ^A,B,C^	1.6%	19.6% ^G,H^	78.8% ^A,B^	3.2%	26.3% ^G,H^	70.5% ^A,B^
	30–39	F	313	1.6%	21.4% ^H^	77.0% ^A,B^	1.3%	20.4% ^H^	78.3% ^A,B,C^	1.3%	15.7% ^H^	83.1% ^A,B,C,D^	2.2%	21.1% ^H^	76.7% ^A,B,C^
	40–49	G	313	2.2%	15.3%	82.4% ^A,B,C,E^	1.6%	21.7%	76.4% ^A,B,C,D^	1.3%	11.5%	87.2% ^A,B,C,D,E^	3.2%	15.3%	81.5% ^A,B,C,D,E^
	50–59	H	312	1.6%	11.9%	86.5% ^A,B,C,D,E,F^	1.6%	11.2%	87.2% ^A,B,C,D,E,F^	1.9%	6.7%	91.3% ^A,B,C,D,E,F^	2.6%	12.8%	84.6% ^A,B,C,D,E,F^

^A,B,C,D,E,F,G,H^ denotes significantly high compared to group A, B, C, D, E, F, G, or H respectively (P<0.05).

**Table 6:** Attitudes towards each type of third-party reproduction assuming the respondents cannot conceive in other ways.

				donor sperm			donor egg			donor embryo			gestational surrogacy		
sex	infertility	group	N	want to use	want to use, if my spouse wishes	not want to use, even if my spouse wishes	want to use	want to use, if my spouse wishes	not want to use, even if my spouse wishes	want to use	want to use, if my spouse wishes	not want to use, even if my spouse wishes	want to use	want to use, if my spouse wishes	not want to use, even if my spouse wishes
		total	2500	1.7%	23.2%	75.1%	1.6%	25.2%	73.2%	1.6%	21.4%	76.9%	2.6%	26.5%	70.9%
male	+	A	122	4.1%	27.9% ^D^	68.0%	5.7% ^B,D^	33.6% ^C,D^	60.7%	4.1% ^D^	28.7% ^C,D^	67.2%	3.3%	35.2% ^C,D^	61.5%
	-	B	1128	1.5%	27.5% ^C,D^	71.0%	1.2%	33.0% ^C,D^	65.8%	1.5%	29.6% ^C,D^	68.9%	2.2%	34.0% ^C,D^	63.7%
female	+	C	236	2.5%	18.6%	78.7% ^A,B^	2.1%	17.4%	80.5% ^A,B^	2.5%	12.3%	85.2% ^A,B^	1.7%	20.3%	78.0% ^A,B^
	-	D	1014	1.5%	18.8%	79.7% ^A,B^	1.5%	17.3%	81.3% ^A,B^	1.3%	13.6%	85.1% ^A,B^	3.1%	18.5%	78.4% ^A,B^

^A,B,C,D^ denotes significantly high compared to group A, B, C, or D respectively (P<0.05).

### Offspring’s right to know their origin

First, we asked the respondents if we should disclose the offspring’s origin to those born via third-party reproduction; 46% of all respondents agreed, 20% disagreed, and 31% were indecisive (Table 7). The frequency of those who agreed was significantly higher in males or females in the 20–29 and 30–39 year age groups than in the 50–59 year age group, and was highest in females in the 20–29 year age group (55.4%). The frequency of those who disagreed was higher in males or females in the 50–59 year age group than in the 20–29 and 30–39 year age groups. In addition, the frequency of those who disagreed was higher in males or females with infertility than in those without infertility (Table 8).

**Table 7:** Attitudes towards offspring’s right to know their origin.

				Offspring has the right to know their origin				Would you receive treatment, even if offspring are granted the right to know their origin?			
sex	age	group	N	agree	disagree	indecisive	others	YES	hesitate or cease	would not receive it originally.	indecisive
		total	2500	46.3%	20.4%	31.7%	1.6%	18.4%	9.5%	39.2%	32.9%
male	20–29	A	312	48.7% ^H^	14.7%	34.6%	1.9%	21.5% ^H^	10.9%	29.8%	37.8% ^D,F,G,H^
	30–39	B	313	49.8% ^H^	18.5%	31.0%	0.6%	21.7% ^H^	9.6%	29.7%	39% ^D,F,G,H^
	40–49	C	313		42.2%	23% ^A,E^	33.5%	1.3%		15.7%	8.9%	37.4%	38% ^D,F,G,H^
	50–59	D	312	43.3%	27.2% ^A,B,E,F,G^	28.5%	1.0%	19.9% ^H^	11.2%	39.4% ^A,B^	29.5%
female	20–29	E	312	55.4% ^C,D,F,G,H^	13.1%	29.5%	1.9%	21.8% ^G,H^	9.6%	33.7%	34.9% ^H^
	30–39	F	313	47.3% ^H^	18.5%	32.9%	1.3%	18.8% ^H^	9.9%	42.2% ^A,B,E^	29.1%
	40–49	G	313	45.7%	18.5%	32.9%	1.3% ^B^	15.3%	6.7%	47.9% ^A,B,C,D,E^	30.0%
	50–59	H	312	37.8%	29.5% ^A,B,E,F,G^	31.1%	1.6%	12.2%	9.3%	53.8% ^A,B,C,D,E,F^	24.7%

^A,B,C,D,E,F,G,H^ denotes significantly high compared to group A, B, C, D, E, F, G, or H respectively (P<0.05).

**Table 8:** Attitudes towards offspring’s right to know their origin.

					Offspring has the right to know their origin					Would you receive treatment, even if offspring are granted the right to know their origin?			
sex	infertility	group	N		agree	disagree	indecisive	others		YES	hesitate or cease	would not receive it originally.	indecisive
		total	2500		46.3%	20.4%	31.7%	1.6%		18.4%	9.5%	39.2%	32.9%
male	+	A	122		45.9%	28.7% ^B,D^	25.4%	0.0%		27% ^B,D^	14.8% ^D^	35.2%	23.0%
	-	B	1128		46.0%	20.0%	32.6% ^C^	1.3%		18.9%^D^	9.7%	34.0%	37.5% ^A,C,D^
female	+	C	236		49.2%	29.7% ^B,D^	19.9%	1.3%		26.7% ^B,D^	12.7% ^D^	41.5% ^B^	19.1%
	-	D	1014		46.0%	17.7%	34.1% ^C^	2.3%		14.8%	8.0%	45.1% ^A,B^	32.1% ^A,C^

^A,B,C,D^ denotes significantly high compared to group A,B,C, or D respectively (P<0.05).

Next, we asked all respondents whether, if they would undergo third party reproductive treatment, they would still do so if origin was disclosed to the offspring. In response, 18.4% said yes, 9.5% would hesitate or cease to receive the treatment, 39.2% would not receive the treatment originally, and 32.9% were indecisive (Table 7). The frequency of those who would receive treatment was significantly lower in females in the 50–59 year age group than in males or females in the 20–29 or 30–39 year age group. The rate of those who would not receive the treatment originally was 33.7%, 42.2%, 47.9%, and 53.8% in females in the 20–29, 30–39, 40–49, and 50–59 year age groups, respectively, showing an increasing tendency with age.

As shown in Table 8, the frequency of those who would receive treatment was significantly higher in males or females with infertility than in those without infertility. In addition, the frequency of those who would hesitate or cease to receive treatment if the offspring were to be informed of their origin was also significantly higher in females with infertility. There was no significant difference between males with or without infertility, although similar trends were observed (14.8% vs. 9.7%). The rate of those who were indecisive was significantly lower in males or females with infertility than in those without.

## Discussion

We conducted a web-based survey on the public attitude towards third-party reproduction and its disclosure to the offspring in Japan. To date, there has been no large-scale survey on the public attitude of Japanese people towards disclosure of third-party reproduction to the offspring. Firstly, approximately 40% of respondents agreed that gamete donation or gestational surrogacy should be approved, whereas 25% answered that it should not be approved. The number of respondents agreeing to third-party reproduction was lowest in females in the 50–59 year age group. Additionally, this number was significantly higher in males or females with infertility, compared to those without, suggesting that individual experience played a role. Secondly, the rate of those who would receive third-party reproduction if their spouse wished to showed a decreasing trend with age. There was no significant difference in the rate of those who would receive third-party reproduction if their spouse wished to between the respondents with or without infertility of both genders. Thirdly, 46.3% of respondents agreed that offspring had the right to know their origin, whereas 20.4% disagreed. Of those who disagreed, the majority were males and females in the 50–59 year age group, and those with infertility. Effects of gender, age, and infertility are important in understanding the attitudes towards third-reproduction and the disclosure to offspring.

The number of respondents accepting of gamete or embryo donation and surrogate pregnancy was similar to that in the survey performed in 2003, suggesting little change in attitudes over 10 years. Furthermore, although the Japan Society of Obstetrics and Gynecology guidelines prohibit surrogacy [4, 9], over 40% of people approve of it. Disapproval of surrogacy was significantly higher in older men, but there was no significant difference in age among females.

The top reason for agreeing with donation was that people who were unable to conceive due to disease or aging might become pregnant. The main reasons for not agreeing with donation were that there was no genetic linkage and that family relations became unnatural. Pregnancies resulting from oocyte donation have reportedly been associated with adverse obstetric and neonatal outcomes [10, 11]; however, these issues were not the main reason for disapproval in this study.

Regarding gestational surrogacy, the top reason for approval was that people who were unable to conceive due to disease, or those who underwent hysterectomy due to disease or accident, could have children. The reasons for disapproval were similar to those for gamete donation. Gestational surrogacy has been reported to increase adverse perinatal outcomes, including preterm birth, low birth weight, hypertension, gestational diabetes, and placenta previa, compared with spontaneous pregnancy by the same women [12]. These were not mentioned, however, as reasons for disapproval in this study. Information on these risks should be supplied. Indeed, a previous report suggested that the disapproval rate of gestational surrogacy was increased by recognizing the associated perinatal adverse outcomes [7].

Respondents were asked if they would choose third-party reproduction if they could not conceive in other ways; the positive opinion frequency was higher in males, in line with previous reports [6, 13, 14]. This was presumed to be due to the fact that men are not physically involved in the procedure and that the desire for descendants is strong [14]. Negative attitudes to all forms of third-party reproduction increased with increasing age. These trends were consistent with previous reports [5]. People with infertility tended to socially approve third-party reproduction more often, but when it came to undergoing the procedures themselves, they were cautious.

With regard to disclose of origin to the offspring born from third-party reproduction, as many as 46% of respondents approved, 20.4% disapproved, and 31.7% were undecided. This prevalence of opinions is similar to the result (38.4%, 29.6%, and 32%, respectively) obtained in Germany [15], where egg donation and reception are prohibited by law. In contrast, 83% of women and 75% of men in Sweden supported the right of offspring to know their origins [16]. This high proportion of approval might relate to the legal liberalization of gamete donation, considering that Sweden is one of the countries permitting oocyte donation and granting offspring the right to know the identity of the donor.

When analyzing the results by age and gender, the frequency of those who approved was lowest in women in their 50s; about one-third of male or female respondents in their 50s opposed it. In addition, there was a significantly higher negative opinion on the offspring’s right to know their origin in men and women with infertility, compared to those without. Furthermore, when the right to know the origin was recognized, there was a significant increase in both those who would continue with infertility treatment and those who would hesitate to do so. As the number of people who answered "cannot decide" significantly reduced in those with infertility, it suggests that people with infertility can form a clear opinion whether they are positive or negative. However, over 30% were indecisive, which might indicate that it is generally difficult to visualize the consequences of different scenarios for recipients and their offspring. These findings may indicate the need for more adequate information and education of the community to enhance the discussion on the ethical consensus on third-party reproduction.

It is necessary to comment on the respondents’ background. Firstly, the enrollment rate of university of is very high among respondents in this study. This reflects that the enrollment rate of Japanese universities originally is more than 50% [17], and we do not regard it as a bias by our study procedure. Secondly, religious background data is lacking. Although we should ask about the religious background in this questionnaire, the Japanese people religious background is homogeneous compared to other country, and most Japanese people answered that they were atheist, according to recent survey [18]. Therefore, the influence of religion might be less in Japan than other countries.

Recently, the number of Japanese infertile patients who have travelled across the border to undergo third-party reproduction has increased. [19]. This is because third-party reproduction is not readily available to infertile Japanese patients. Several papers point out that a law or regulation on third-party reproduction should be made [4, 9]. Semba et al. indicated that Japan will merely export ethical issues to a permissive country if the banning of surrogacy in Japan results in more infertile patients seeking treatment abroad [4].

Very recently, there have been several cases of birth after uterus transplantation [20–22]. Uterine transplantation has not been carried out in Japan; however, a positive result has been obtained in a public survey on uterus transplantation when compared to surrogacy in Japan [23]. Although uterine transplantation is still in an experimental phase, it may be an option and may have the potential to change the ethical viewpoints and attitudes towards gestational surrogacy.

The present study has certain limitations. First, this study is a cross-sectional study, which cannot explore a causal relationship. Second, there is the possibility of survey selection bias and issues of generalizability. After reading the questionnaire introduction, respondents had the choice whether to complete the survey or not. Third, we have not confirmed the level of knowledge and understanding on third-party reproduction of the respondents. Despite these limitations, by conducting a web-based questionnaire of the general population, we collected a large sample size; therefore, the findings of this study reflect the public attitudes towards third-party reproduction in Japan.

## Conclusion

In the current study, we clarified that the public attitudes towards third-party reproduction and the disclosure to offspring were affected by gender, age, and the experience of infertility. Respondents who were indecisive numbered over 30%, which might indicate the requirement for more information and education of the community to enhance the discussion on the ethical consensus on third-party reproduction. Investigating and gaining knowledge regarding the general attitudes towards these issues is important. Legal and medical professionals require this type of research in order to make predictions about behaviors in the future, and to allow regulation or legislation concerning third-party reproduction.

## Supporting information

S1 Fig Reference materials to deepen the understanding of respondents. (PPTX)

S1 Document The questionnaire used in this study. (DOCX)

S1 Table The demographic data of each group respondents (N = 2500). (XLSX)

S2 Table Why do you think that we should approve in vitro fertilization with the provision of a third-party egg, sperm, or embryo? (XLSX)

S3 Table Why do you think that in vitro fertilization using sperm, egg, embryo of a third party is not socially acceptable? (XLSX)

S4 Table Why do you think that we should approve a surrogate pregnancy using the womb of a third party? (XLSX)

S5 Table Why do you think that surrogate pregnancy using a third party's uterus is not socially acceptable? (XLSX)
