# Supplementary material for: A survey of public attitudes towards third-party reproduction in Japan in 2014
Source: PLoS One. 2018 Oct 31;13(10):e0198499. doi: 10.1371/journal.pone.0198499 (PMC6209135; doi:10.1371/journal.pone.0198499)
Supplement: S1 Fig — (PPTX) [file pone.0198499.s001.pptx]

## Slide 1
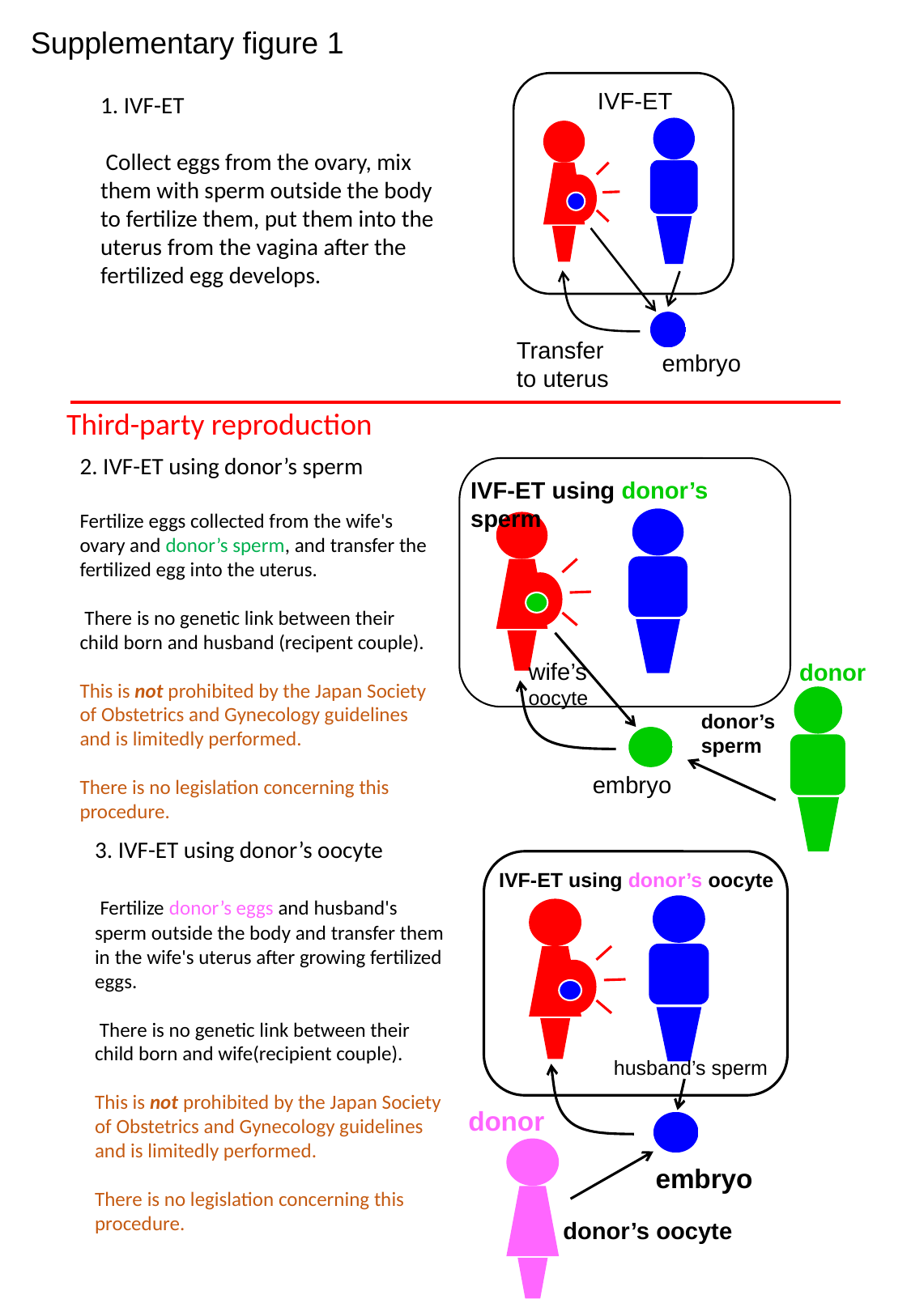

Supplementary figure 1
 IVF-ET
Transfer to uterus
embryo
1. IVF-ET
 Collect eggs from the ovary, mix them with sperm outside the body to fertilize them, put them into the uterus from the vagina after the fertilized egg develops.
Third-party reproduction
2. IVF-ET using donor’s sperm
Fertilize eggs collected from the wife's ovary and donor’s sperm, and transfer the fertilized egg into the uterus.
 There is no genetic link between their child born and husband (recipent couple).
This is not prohibited by the Japan Society of Obstetrics and Gynecology guidelines and is limitedly performed.
There is no legislation concerning this procedure.
IVF-ET using donor’s sperm
embryo
wife’s oocyte
donor
donor’s sperm
3. IVF-ET using donor’s oocyte
 Fertilize donor’s eggs and husband's sperm outside the body and transfer them in the wife's uterus after growing fertilized eggs.
 There is no genetic link between their child born and wife(recipient couple).
This is not prohibited by the Japan Society of Obstetrics and Gynecology guidelines and is limitedly performed.
There is no legislation concerning this procedure.
IVF-ET using donor’s oocyte
donor
embryo
donor’s oocyte
husband’s sperm

## Slide 2
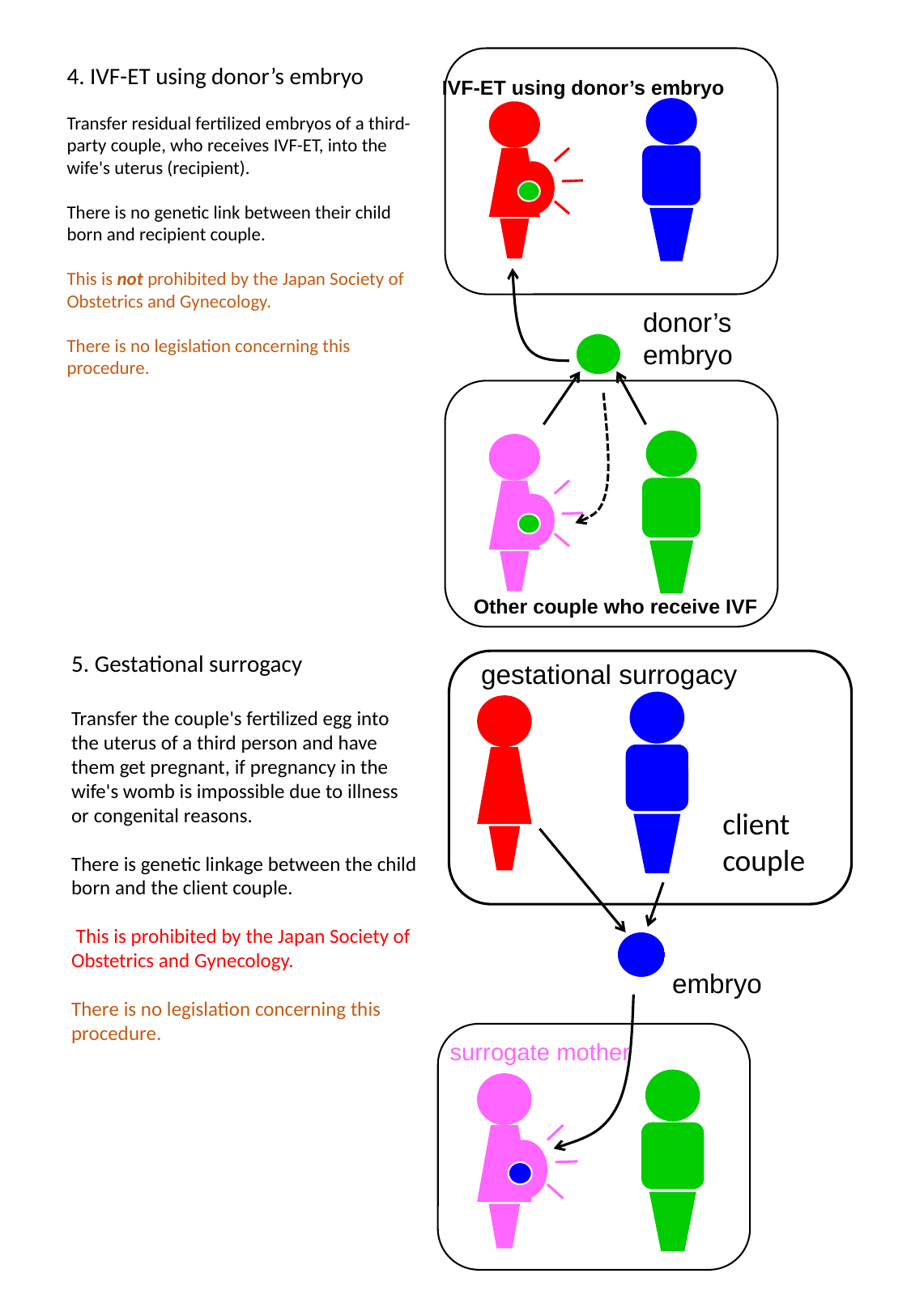

donor’s embryo
IVF-ET using donor’s embryo
4. IVF-ET using donor’s embryo
Transfer residual fertilized embryos of a third-party couple, who receives IVF-ET, into the wife's uterus (recipient).
There is no genetic link between their child born and recipient couple.
This is not prohibited by the Japan Society of Obstetrics and Gynecology.
There is no legislation concerning this procedure.
Other couple who receive IVF
5. Gestational surrogacy
Transfer the couple's fertilized egg into the uterus of a third person and have them get pregnant, if pregnancy in the wife's womb is impossible due to illness or congenital reasons.
There is genetic linkage between the child born and the client couple.
 This is prohibited by the Japan Society of Obstetrics and Gynecology.
There is no legislation concerning this procedure.
gestational surrogacy
embryo
surrogate mother
client couple
