# Supplementary material for: A survey of public attitudes towards third-party reproduction in Japan in 2014
Source: PLoS One. 2018 Oct 31;13(10):e0198499. doi: 10.1371/journal.pone.0198499 (PMC6209135; doi:10.1371/journal.pone.0198499)
Supplement: S1 Document — (DOCX) [file pone.0198499.s002.docx]

Questionnaire title: Questionnaire on reproductive assistance technology involving third parties

【Q.1】 What is your gender?

1. Men

2. Women

【Q.2】 What is your age?

( ) year old

【Q.3】 Are you married?

* For those who lost partner or has been divorced, please answer "1. unmarried".

1. Unmarried

2. Married

【Q.4】 How many children do you have?

1. No children

2. One

3. Two

4. Three

5. Four

6. More than 4.

【Q.5】 How much is your annual household income ?

1. Less than 1 million yen/year

2. 　1 ~ 3 million yen/year

3. 　3 ~ 5 million yen/year

4. 　5 ~ 7 million yen/year

5. 　7 ~ 10 million yen/year

6. 10 ~ 15 million yen/year

7. 15 million yen or more/year

【Q.6】 Which school did you graduate last?

1. Junior High School

2. High school

3. Technical College, Vocational School

4. Junior college

5. University or graduate school

【Q.7】 Do you have any experiences suffering from infertility ?

1. I have experience

2. I have no experience

【Q.8】 Have you ever received the following infertility treatment?

1. Timed intercourse

2. IUI (intrauterine insemination)

3. IVF-ET (in vitro fertilization – embryo transfer)

4. ICSI (intracytoplasmic sperm injection)

5. I have never undergone infertility treatment

【Q.9】 If you desire a child, and if you do not have children, do you want to use the following technology?

A. In vitro fertilization using third-party sperm

1. I want to use

2. I would like to use it if my spouse wishes

3. I do not use it even if my spouse wishes

B. In vitro fertilization using a third-party egg

1. I want to use

2. I would like to use it if my spouse wishes

3. I do not use it even if my spouse wishes

C. Embryo transplantation using third party fertilized eggs (embryos)

1. I want to use

2. I would like to use it if my spouse wishes

3. I do not use it even if my spouse wishes

D. Gestational surrogacy using a third-party uterus

1. I want to use

2. I would like to use it if my spouse wishes

3. I do not use it even if my spouse wishes

【Q.10】 Which do you think is socially unacceptable?

1. In vitro fertilization using sperm / egg / fertilized egg (embryo) of a third party should not be approved.

2. Gestational surrogacy using third-party uterus should not be approved

3. Neither should be approved

4. Both of them should be approved.

5. indecisive

<Respondent: person who selected 1or 3 with Q10>

【Q.11】 Why do you think that "in vitro fertilization using sperm, egg, embryo of a third party is ***not*** socially acceptable"?

1. Because there is a possibility of harm to the health of the child born.

2. Because there is no genetic link with parents.

3. I think that family relationship (parent-child) relationship will become unnatural

4. Because I think that pregnancy should be natural.

5. When a child born is married, there is a possibility of consanguineous marriage

6. I think that it will be used commercially.

7. Because I cannot tell my children that they were born with in vitro fertilization using sperm / egg / embryos of a third party

8. Because there are other options such as adoption.

9. Because we use people as reproductive means.

10. Other

11. No idea

<Respondent: person who selected 2or 3 with Q 10, MA>

【Q.12】 Why do you think that "surrogate pregnancy using a third party's uterus is ***not*** socially acceptable"?

1. Because there is a possibility of harm to the health of the child born.

2. I think that family relationship (parent-child) relationship will become unnatural

3. Because there is a possibility that various troubles such as parental authority and inheritance problem will occur.

4. I think that pregnancy should be natural.

5. I think that it will be used commercially.

6. Because I cannot tell my child that I was born in a surrogate pregnancy using a third-party uterus.

7. Because there are other options such adoption.

8. Because we use people as reproductive means.

9. Other

10. No idea

<Respondent: Person who selected 2or 4 with Q10>

【Q.13】 If in vitro fertilization using a third-party egg, sperm, fertilized egg (embryo) is recognized socially, who is eligible to be a gamete or embryo recipient?

1. Married couple

2. Fact-married couple, cohabiting couple

3. Single women

4. Single men

5. Homosexual couple

6. Other

7. No idea

<Respondent: Person who selected 2or 4 with Q10>

【Q.14】Why do you think that we should approve in vitro fertilization with the provision of a third party egg, sperm, or embryo?

1. Because there is a possibility that a person who cannot conceive because of illness can have a child.

2. People who are unable to conceive because of aging will be able to be pregnant.

3. Homosexual couples can have children.

4. Because there is a possibility that a single person can have a child.

5. It is because both the client and the donor agreed.

6. Other

7. No idea

<Respondent: Person who selected 1 or 4 in Q10>

【Q.15】 Why do you think that we should approve a surrogate pregnancy using a third-person's uterus?

1. Because there is a possibility that a person who cannot conceive because of illness can have a child.

2. Because there is a possibility that a woman who removed the uterus due to disease or accident can have a child.

3. Because women who cannot be pregnant due to her work circumstances have the possibility of having children.

4. Since homosexual couples may have children,

5. Because there is a possibility that a single person can have a child.

6. Because both the client and the surrogate mother agreed.

7. Others

8. No idea

<About the right to know the origin of children born in assisted reproductive medicine>

【Q.16】 How do you think about the fact that a child born from a third-party sperm, egg, or embryo, or a child born by gestational surrogacy, has the right to know about it?

1. Agree

2. Disagree.

3. Indecisive

4. Others

【Q.17】 Would you receive treatment, even if offspring are granted the right to know their origin?

1. Yes, I would receive the treatment.

2. I hesitate or cease to receive the treatment.

3. I would not receive it originally.

4. Indecisive
